# Electrographic seizures after subarachnoid hemorrhage lead to derangement of brain homeostasis in humans

**DOI:** 10.1186/cc9751

**Published:** 2011-03-11

**Authors:** J Claassen, A Perotte, D Albers, J Schmidt, B Tu, N Badjatia, K Lee, S Mayer, E Connolly, L Hirsch, G Hripcsak

**Affiliations:** 1 Columbia University, New York, USA

## Introduction

This study intends to develop a physiologic thumbprint for nonconvulsive seizures (NCSz) after acute brain injury. Abnormal electrographic brain activity including NCSz is common after acute brain injury and is associated with poor outcome. Mechanisms underlying this phenomenon are poorly understood but in animals periods of inadequate perfusion during seizures have been documented. In the present study we hope to gain better understanding of the relationship between abnormal electrographic patterns and brain homeostasis in patients with subarachnoid hemorrhage (SAH).

## Methods

Between June 2006 and June 2010, 51 poor-grade SAH patients underwent multimodality monitoring with microdialysis, brain oxygen tension (pbtO_2_), regional cerebral blood flow (rCBF), and intracranial pressure monitoring; 69% (*n *= 36) also with intracortical EEG (ICE; eight-contact miniature depth electrode). Each minute of EEG (total of 326,513 minutes) was categorized separately into non-ictal, on the ictal-interictal continuum (including periodic discharges at 2 Hz or faster), or seizures. We identified seizure onsets on ICE recordings and extracted the physiologic monitoring data 30 minutes pre and post seizure onset. Physiologic profiles based on standard error of the means plots were generated using high-frequency time series physiologic measurements and interpreted by visual analysis.

## Results

Depth NCSz were recorded in 36% (13/36) of patients with ICE recordings (depth seizures in 11,017 minutes). NCSz were preceded by an increase in rCBF starting 15 minutes prior to onset of depth NCSz that stayed elevated throughout the observation period. Heart rate, mean arterial, intracranial, and cerebral perfusion pressures were elevated surrounding NCSZ. There was a small transient drop in PbtO_2 _and a drop in jugular bulb oxygen saturation seen between 1 and 3 minutes following seizure onset. There was a small rise in brain temperature but no change in bladder temperature associated with the NCSZs, but water temperature of the cooling device dropped following seizure onset.

## Conclusions

These findings confirm in comatose human beings that NCSz detected by ICE are associated with hyperemia, increased metabolism, and possibly brain tissue hypoxia, which serve as surrogates for secondary brain injury. Future research should implement novel approaches for ICU time-series data analysis, evaluate surface seizures, and utilize other surrogates of brain metabolism such as microdialysis.

